# Effects of hepatocyte nuclear factor-1A and -4A on pancreatic stone protein/regenerating protein and C-reactive protein gene expression: implications for maturity-onset diabetes of the young

**DOI:** 10.1186/1479-5876-11-156

**Published:** 2013-06-26

**Authors:** Ma P Kyithar, Caroline Bonner, Siobhan Bacon, Seán M Kilbride, Jasmin Schmid, Rolf Graf, Jochen HM Prehn, Maria M Byrne

**Affiliations:** 1 Department of Endocrinology, Mater Misericordiae University Hospital, 30 Eccles Street, Dublin 7, Ireland; 2 Department of Physiology and Medical Physics and Centre for Systems Medicine, Royal College of Surgeons in Ireland, Dublin 2, Ireland; 3 Department of Visceral and Transplantation Surgery, University Hospital, Zurich, Zurich, Switzerland; 4 Current address: Biotherapies for Diabetes, Univ Lille Nord de France, F-59000, Lille, France

**Keywords:** HNF1A, HNF4A, MODY, PSP/reg, HsCRP, Gene regulation

## Abstract

**Background:**

There is a significant clinical overlap between patients with hepatocyte nuclear factor (HNF)-1A and HNF4A maturity-onset diabetes of the young (MODY), two forms of monogenic diabetes. HNF1A and HNF4A are transcription factors that control common and partly overlapping sets of target genes. We have previously shown that elevated serum pancreatic stone protein / regenerating protein A (PSP/reg1A) levels can be detected in subjects with HNF1A-MODY. In this study, we investigated whether PSP/reg is differentially regulated by HNF1A and HNF4A.

**Methods:**

Quantitative real-time PCR (qPCR) and Western blotting were used to validate gene and protein expression in cellular models of HNF1A- and HNF4A-MODY. Serum PSP/reg1A levels and high-sensitivity C-reactive protein (hsCRP) were measured by ELISA in 31 HNF1A- and 9 HNF4A-MODY subjects. The two groups were matched for age, body mass index, diabetes duration, blood pressure, lipid profile and aspirin and statin use.

**Results:**

Inducible repression of HNF1A and HNF4A function in INS-1 cells suggested that *PSP/reg* induction required HNF4A, but not HNF1A. In contrast, *crp* gene expression was significantly reduced by repression of HNF1A, but not HNF4A function. PSP/reg levels were significantly lower in HNF4A subjects when compared to HNF1A subjects [9.25 (7.85-12.85) ng/ml vs. 12.5 (10.61-17.87) ng/ml, U-test *P* = 0.025]. hsCRP levels were significantly lower in HNF1A-MODY [0.22 (0.17-0.35) mg/L] compared to HNF4A-MODY group [0.81 (0.38-1.41) mg/L, U-test *P* = 0.002], Parallel measurements of serum PSP/reg1A and hsCRP levels were able to discriminate HNF1A- and HNF4A-MODY subjects.

**Conclusion:**

Our study demonstrates that two distinct target genes, *PSP/reg* and *crp*, are differentially regulated by HNF1A and HNF4A, and provides clinical proof-of-concept that serum PSP/reg1A and hsCRP levels may distinguish HNF1A-MODY from HNF4A-MODY subjects.

## Background

Maturity-onset diabetes of the young (MODY) is an autosomal dominant form of non-ketotic, non-insulin dependent diabetes that is typically diagnosed before 25 years of age. The majority of MODY subjects are defined by mutations in six specific MODY-related genes, including the nuclear transcription factors hepatocyte nuclear factor (HNF)-1A and HNF-4A [1-3]. HNF1A-MODY represents the most common form of MODY [3-5]. Mutations in *HNF4A* are less common than mutations in *HNF1A*, however over 30 mutations have been identified to date [6]. *HNF1A* and *HNF4A* mutations cause a similar clinical phenotype of MODY, characterized by progressive beta-cell dysfunction, defects in glucose-stimulated insulin secretion [7,8] and sensitivity to low-dose sulphonylureas [9]. However infants with *HNF4A* mutations are at risk of developing macrosomia and transient as well as persistent hyperinsulinaemic hypoglycaemia [10,11]. Hence specific biomarkers that would differentiate between *HNF1A* and *HNF4A* mutations would facilitate better identification of these subtypes. In addition, features of *HNF1A* and *HNF4A* mutation carriers tend to overlap with type 1 diabetes, type 2 diabetes and other monogenic forms of diabetes [12,13]. Non-specific clinical features of MODY result in difficulty in selecting the appropriate molecular testing [13]. Sequencing is considered the standard method for mutation detection in individuals with monogenic diabetes. However sequencing is expensive and not immediately available in many hospitals.

HNF1A and HNF4A belong to the steroid/thyroid hormone receptor superfamily of transcription factors [14]. HNF1A and HNF4A act in a transcription factor network with HNF4A controlling the activity of HNF1A [15]. This network plays a fundamental role in the early development of the pancreas, liver and intestine [16]. Both transcription factors are also important for the maintenance of beta-cell function throughout life, and influence the expression of *insulin* and the principle glucose transporter *Glut2,* among many other target genes [14,17-20]. Molecular studies have demonstrated that HNF4A-regulated gene expression patterns are remarkably similar to that of its downstream transcription regulatory protein, HNF1A, and that the two factors may activate transcription by a synergistic action [21-23]. We have previously demonstrated that elevated serum pancreatic stone protein / regenerating protein A (PSP/reg1A) levels can be detected in subjects with HNF1A-MODY compared to HNF1A-MODY-negative non-diabetic family members [24]. We have also shown that PSP/reg1A levels did not correlate with hyperglycemia [25]. Other studies have identified high-sensitivityC-reactive protein (hsCRP), a known HNF1A target gene [26,27] to be reduced in serum levels of subjects with HNF1A-MODY, compared to other forms of diabetes such as type 1 diabetes, type 2 diabetes, HNF4A-MODY, and glucokinase-MODY [28-30]. The concept that the *crp* gene is not regulated by HNF4A, despite HNF1A being downstream of HNF4A, has not yet been mechanistically proven, and there is evidence for a partial overlap of hsCRP levels in HNF1A- and HNF4A-MODY subjects [29,30]. Furthermore, hsCRP levels are elevated during inflammation, demonstrating the need for additional biomarkers. In this study we aimed (i) to investigate whether HNF1A and HNF4A differentially influence the expression of *PSP/reg* and *crp*, and (ii) to provide clinical proof-of-concept that parallel measurements of PSP/reg1A and hsCRP levels may be of clinical use in distinguishing HNF1A- from HNF4A-MODY subjects.

## Materials and methods

### Inducible repression of HNF1A and HNF4A function in insulinoma INS-1 cells

Rat INS-1 insulinoma cells overexpressing a dominant-negative (DN) mutant of HNF1A-MODY (Pro291fsinsC-HNF1A), carrying a C nucleotide insertion in the polyC-tract that results in the translation of a truncated dominant-negative protein, or expressing a dominant negative mutant of HNF4A (DN-HNF4A), lacking the first 111 amino acids, all under the control of a doxycycline-dependent transcriptional activator have been described previously [31-36]. Cells were cultured in RPMI 1640 medium supplemented with 10% FBS (PAA, Cölbe, Germany), 2 mmol/l L- glutamine, 1 mmol/l pyruvate, penicillin (100 U/ml), streptomycin (100 μg/ml), 10 mmol/l HEPES (pH 7.4) and 50 μmol/l 2-mercaptoethanol (Sigma, Dublin, Ireland) [37]. For experiments, cells were seeded at a density of 5 × 10^4^ cells/cm^2^ for 48 h prior to treatment with / without doxycycline (500 ng/ml), and were cultured in RPMI 1640 medium containing 6 mmol/liter glucose and co-treated with reagents as indicated.

### Real-time quantitative RT-PCR (qPCR)

Expression patterns of *Glut2*, *insulin, Crp*, and *PSP/reg* mRNA were examined using real-time qPCR. INS-1 cells were harvested from culture treatments at the appropriate time-points. Total RNA was extracted using the RNeasy mini Kit (Qiagen, Crawley, UK). First-strand cDNA synthesis was performed using 2 μg total RNA as template and Superscript II reverse transcriptase (Invitrogen) primed with 50 pmol random hexamers (New England Bio labs, Ipswich, MA, USA). Quantitative real-time PCR was performed using the Light Cycler 2.0 (Roche Diagnostics, Indianapolis, IN, USA) and the QuantiTech SYBR Green PCR kit (Qiagen) as per manufacturers’ protocols and 25 pmol of primer pair concentration (Sigma-Genosys).

Specific PCR products (150–250 bp in length) for each gene analysed were designed using Primer3 software (http://bioinfo.ut.ee/primer3-0.4.0/) [38]. Sense and antisense primers were designed for all primers

AACAGCACCTTTGTGGTCCT and GTGCAGCACTGATCCACAAT for *insulin;*

CAATTTCATCATCGCCCTCT and GTCTCTGATGACCCCAGGAA for *Glut2;*

GGCTTTGACGCGAATCAGTC and AGTCAGTCAAGGGCCACAGC for *Crp;*

AGGCCAGGAGGCTGAAGAAG and TGGAGGCCAATCCAGACATT for *PSP/reg;*

and AGCCATCCAGGCTGTGTTGT and CAGCTGTGGTGGTGAAGCTG for *β-actin.*

### Western blotting

Cells were rinsed with ice-cold phosphate-buffered saline (PBS) and scraped before being lysed in buffer containing 62.5 mM Tris-Cl (pH 6.8), 2% SDS, 10% glycerin and protease inhibitor cocktail (Sigma, Dublin, Ireland). Protein content was determined using the Pierce BCA Micro Protein Assay kit (Thermo Fisher Scientific Inc., Rockford, Illinois). Samples were supplemented with 2-mercaptoethanol (Sigma Aldrich, Dublin, Ireland) and denaturated at 95°C for 5 min. An equal amount of protein (25–50 μg) was separated with 12–15% SDS-PAGE and blotted to nitrocellulose membranes (Protean BA 85; Schleicher & Schuell, Dassel, Germany). The membranes were blocked with 1% bovine serum albumin in Tris-buffered saline (20 mM Tris, pH 7.5, 150 mM NaCl) for 2 h at room temperature. The primary rabbit polyclonal CRP antibody (Abcam, Cambridge, UK), rabbit polyclonal anti-PSP/*reg* antibody [39] and primary mouse monoclonal β-actin mouse antibody were diluted 1:10,000 in 5% milk (Sigma). The primary mouse monoclonal anti-tubulin antibody (Sigma) was diluted 1:5,000. Antibodies were incubated overnight at 4 C. The membranes were washed in Tris-buffered saline containing 0.05% Tween 20. The secondary antibodies peroxidase-coupled goat anti-rabbit IgG (Sigma) or goat anti-mouse (Jackson Immunoresearch, Europe Ltd) were diluted 1:25,000 in the same buffer. The membranes were washed in Tris-Buffered Saline containing 0.05% Tween 20. Antibody-conjugated peroxidase activity was visualized using the Super Signal chemiluminescence reagent (Pierce, Buckinghamshire, UK). Densitometry was carried out by quantifying the intensities of bands using ImageJ 1.41o. Regions were drawn around each band and the integrated intensity was measured, with intensity of a background region of the same size on the same gel subtracted. The intensity of each protein of interest was divided by the corresponding loading control protein.

### Subjects

Subjects with a clinical diagnosis of MODY were recruited from the diabetes clinics in the Mater Misericordiae University Hospital Dublin in Ireland. Sequencing of the *HNF1A* and *HNF4A* genes were performed by IntegraGen (Bonn, Germany) in 2006–2007 and the Molecular Genetics Laboratory (Exeter, UK) in 2008–2010. Genetically confirmed MODY patients included 33 cases with *HNF1A* mutations and 9 with *HNF4A* mutations. The subjects with *HNF1A* mutations were from 11 pedigrees and the mutations included L17H, G207D, P291finsC, S352fsdelG, F426X, P379T, and IVS7-6G > A. The genotype/phenotype of these patients was published previously, and all mutations described co-segregated with diabetes in all pedigrees [40]. Subjects with *HNF4A* mutations were from 2 pedigrees and the mutations included M1? and R290C. The genotype/phenotype of these two pedigrees was published previously and both mutations likewise co-segregated with diabetes [40]. All subjects underwent full clinical assessment including a full medical history and physical examination. Details of the subjects’ weight, height and blood pressure were recorded. Plasma glucose was measured using Beckman Synchron DXC800 (Beckman Instruments Inc, Brea, USA). Haemoglobin A_1c_ (HbA_1c_) was determined by high-performance liquid chromatography (Menarini HA81-10, Rome, Italy). The study was approved by the Research Ethics Committee at the Mater Misericordiae University Hospital Dublin and all subjects gave informed written consent.

### Measurement of serum PSP/reg1A levels

The enzyme-linked immunosorbent assay (ELISA) was used to quantify human PSP/reg1A. Recombinant human PSP/reg1A protein was used to immunize rabbits and guinea pigs to obtain Antisera [24,39,41]. Serum was prepared by centrifugation, and the IgG were purified by affinity chromatography on protein A columns. Subsequently, a sandwich ELISA was designed on 96-well ELISA plates. Antibody of the first species (Guinea pig) was coated to the bottom, blocked with bovine serum albumin and aliquots of serum were then incubated for two hours. After washing the wells, antibodies of the other species (Rabbit) were incubated, followed by a series of washing steps. Finally, a phosphatase-coupled anti-rabbit IgG was used. The reaction of the phosphatase with a substrate was determined on a multiplate reader (Dynatech) and subjects’ serum PSP/reg1A levels were compared with standard amounts of recombinant human PSP/reg1A protein. PSP/reg1A levels of some of the HNF-1A-MODY subjects are historic data and have been published previously [24,25].

### Measurement of serum hsCRP levels

Serum hsCRP levels were measured using particle enhanced immunonephelometry assay (Cardio Phase® hsCRP, Siemens) on a Siemens BN II analyzer (Siemens Healthcare Diagnostics, Deerfield, IL, USA). A typical limit for detection of hsCRP was 0.175 mg/L for measurements performed using a sample dilution of 1:20. A coefficient of variation at the concentration 0.41 mg/L was 7.6%. We considered that hsCRP values >10 mg/l were likely to represent an inflammatory response in line with previous studies [28,42,43]. We therefore performed two separate analysis approaches, one in which we included (termed ‘all patients’), and one in which we excluded (termed ‘without extreme CRP’) the 2 HNF1A-MODY patients with serum hsCRP values of >10 mg/l.

### Statistical analysis

Clinical data are expressed as median and interquartile range (IQR). Biochemical data are expressed as mean ± standard error of the means (SEM). Statistical analysis was performed using SPSS statistical software package for Windows, version 18.0 (SPSS, Chicago, IL, USA). The significance of the difference between 2 groups was determined by Mann–Whitney U test (non-parametric clinical data) or t test as indicated. For comparisons of categorical data, chi-square test was applied. The Spearman correlation test was used for correlation analysis. Differences and correlations were considered significant at P < 0.05.

In order to investigate the performance of hsCRP and PSP/reg1A to distinguish HNF1A- from HNF4A-MODY subjects, plots of the receiver operating characteristic (ROC) were analyzed and linear discriminant analysis (LDA) was performed. ROC plots were obtained by calculating all sensitivity and specificity pairs for the levels of serum markers observed in the subjects. An observed value reaching similarly high sensitivity and specificity qualified as final cut-off value for classification. LDA was applied to calculate the best equation for a linear cut-off function to discriminate between groups. Both approaches were applied to each serum marker individually and to the ratio of PSP/reg1A to hsCRP to investigate whether a combination of the markers improved discrimination. ROC, LDA and resulting sensitivity and specificity where assessed using Mat Lab R7.4 (The Math works Inc., Natick, MA, USA).

## Results

### Serum PSP/reg levels may discriminate HNF1A-MODY from HNF4A-MODY subjects

**Figure 1:**
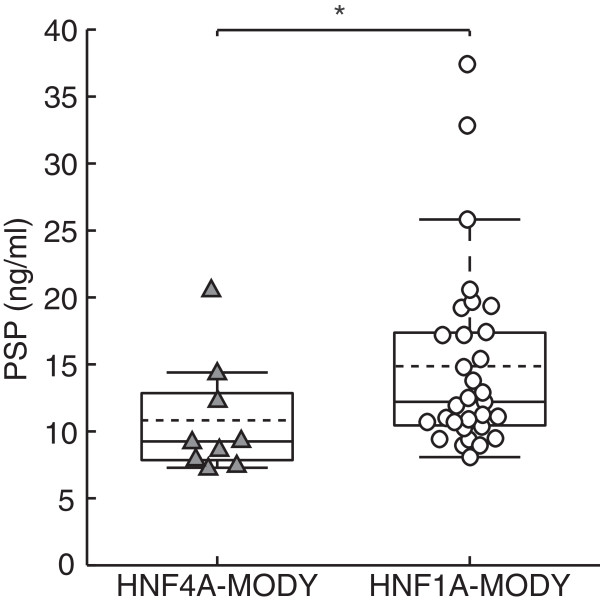
**Serum levels of PSP/reg1A in HNF4A-MODY (▲) versus HNF1A-MODY (○).** Solid lines/box plot showing median and interquartile range and dotted line showing mean. Median of PSP/reg1A in HNF1A-MODY is 9.25 ng/ml (IQR = 7.85-12.85 ng/ml, n = 9) while median in HNF1A-MODY is 12.50 ng/ml (IQR = 10.61-17.87 ng/ml, n = 31). The distributions differ significantly (*): Mann–Whitney U-test p = 0.025.

In a previous study, we have shown that serum levels of the regenerative protein, PSP/reg1A, were elevated in subjects with HNF1A-MODY when compared to MODY-negative body mass index (BMI)-, sex-, and age-matched family members [24]. We were therefore also interested to determine whether HNF4A-MODY carriers showed similarly elevated serum levels of PSP/reg. Clinical characteristics of HNF1A and HNF4A-MODY subjects are shown in Table 1. HNF1A-MODY and HNF4A-MODY subjects were matched for age, BMI, blood pressure, aspirin and statin use, and white cell and neutrophil counts. HbA_1c_ values were moderately higher in HNF1A-MODY subjects. We have previously shown that PSP/reg1A levels did not correlate with HbA_1c_ in HNF1A-MODY [25]. There was no correlation between PSP/reg1A and HbA_1c_ levels in HNF4A-MODY (rho = 0.56, *P* = 0.37). There were no significant differences in duration of diabetes, lipid profile, and fasting plasma glucose between HNF1A-MODY and HNF4A-MODY groups. Interestingly, we found that serum PSP/reg1A levels of HNF4A-MODY subjects were significantly lower than in the HNF1A-MODY subjects [9.25 (7.85-12.85) ng/ml vs. 12.5 (10.61-17.87) ng/ml, U-test *P* = 0.025] (Figure 1). Because PSP/reg1A levels have been shown to be elevated in HNF1A-MODY as well as type 1 diabetes patients [24], this suggested that PSP/reg1A levels may be negatively regulated by HNF4A.

**Table 1:** Clinical characteristics of HNF1A- and HNF4A-MODY subjects

	**HNF1A-MODY median (IQR)**	**HNF4A-MODY median (IQR)**	**P value**
**n (male/female)**	33 (14/19)	9 (4/5)	0.91 NS
**Age (years)**	38 (21–51)	32 (23–46)	0.57 NS
**Duration of diabetes (years)**	6 (3–18)	7 (0–16)	0.42 NS
**Body mass index (kg/m**^**2**^**)**	24.4 (22.0–26.2)	23.9 (21.8–25.0)	0.55 NS
**Systolic blood pressure (mmHg)**	122 (116–131)	115 (109–129)	0.27 NS
**Diastolic blood pressure (mmHg)**	71 (67–80)	73 (67–79)	0.83 NS
**Total cholesterol (mmol/L)**	4.3 (3.6–4.9)	3.7 (3.4–4.3)	0.12 NS
**LDL cholesterol (mmol/L)**	2.5 (2.1–3.0)	2.2 (1.8–2.7)	0.14 NS
**HDL cholesterol (mmol/L)**	1.3 (1.1–1.7)	1.2 (0.8–1.6)	0.28 NS
**TG (mmol/L)**	0.71 (0.55–0.92)	0.62 (0.52–0.79)	0.23 NS
**HbA**_**1c **_**(%)**	7.1 (6.3–8.0)	5.7 (5.3–7.1)	0.045 *
**Fasting plasma glucose (mmol/L)**	6.8 (5.3–8.6)	5.0 (3.8–7.2)	0.09 NS
**WCC (x10**^**9**^**/L)**	6.76 (5.62–8.52)	6.66 (5.94–7.89)	0.71 NS
**Neutrophil count (x10**^**9**^**/L)**	4.06 (3.30–5.73)	3.70 (3.07–4.47)	0.21 NS
**Patients treated with aspirin (n)**	10	2	0.63 NS
**Patients treated with statin (n)**	8	1	0.40 NS

Results are expressed in median and interquartile range (IQR). *: significant; NS: not significant; LDL/HDL: low-density/high-density lipoprotein; TG: triglycerides; WCC: white cell count; Mann–Whitney *U* test for HNF1A-MODY vs. HNF4A-MODY revealed no statistically significant differences but for HbA_1c_. Male/female ratio and treatment with aspirin or statins were tested for statistical difference using the χ^2^ test.

### Inhibition of HNF4A, but not HNF1A function decreases *PSP/reg* gene expression in INS-1 insulinoma cells

**Figure 2:**
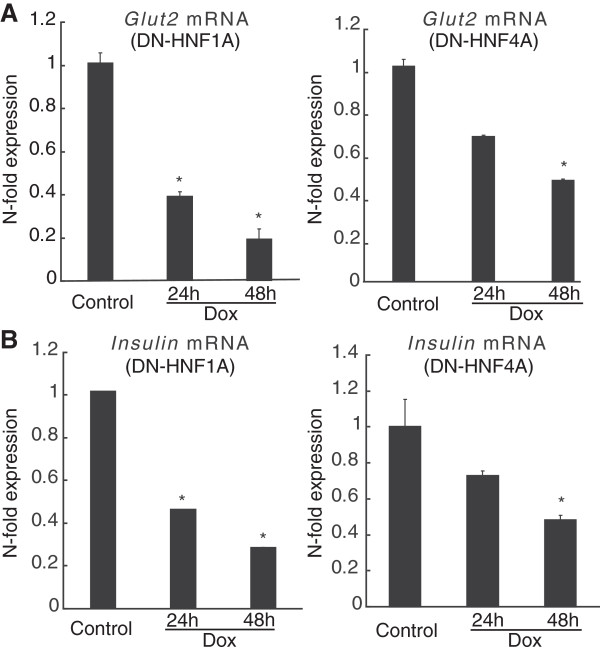
**Effect of suppression of *Hnf1a *and *Hnf4a *function on *Glut2 *and *insulin *expression in INS-1 cells.** DN-HNF1A and DN-HNF4A in INS-1 cells were induced with doxycycline for 0 to 48 h. *Glut2* mRNA expression **(A)** and *insulin* mRNA expression **(B)** were determined using real-time qPCR. Experiments were carried out in triplicate three times, and normalized to β-actin. Data are represented as mean ± SEM. *p < 0.05.

To provide a molecular validation of these clinical findings, we turned to a cellular model of MODY where suppression of HNF1A or HNF4A function in INS-1 cells is achieved through the expression of DN-HNF1A or DN-HNF4A mutants [21][33]. The HNF1A and HNF4A dominant-negative mutants were inducibly expressed in INS-1 cells using a tetracycline- dependent transactivator system through the addition of doxycycline for 24 and 48 h. Suppression of either HNF1A or HNF4A function induced a significant reduction in mRNA levels for *Glut2*, a known HNF1A [33] and HNF4A target gene [21] (Figure 2A). In agreement with previous findings [21][31,33], *insulin* mRNA levels were also significantly reduced during suppression of HNF1A or HNF4A function (Figure 2B).

**Figure 3:**
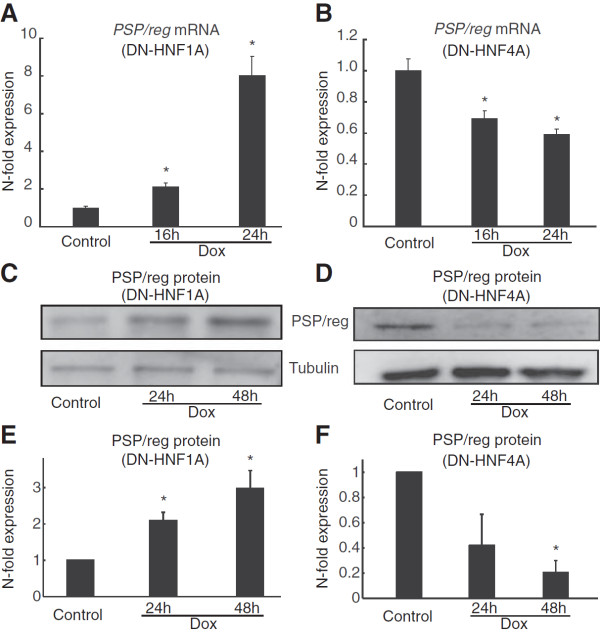
**A Quantification of *PSP/reg *gene expression following inhibition of HNF1A and HNF4A function in INS-1 cells.** INS-1 cells were treated with 500 ng /ml doxycycline from 0 to 48 h to induce DN-HNF1A and DN-HNF4A respectively. mRNA expression of *PSP/reg* was examined using real-time qPCR relative to β-actin. Expression levels were normalized to control cells and data represent means ± SEM from *n* = 3 cultures.* p < 0.05 difference from non-induced controls. Experiments were repeated 3 times with similar results **(A, B)** Whole cell lysates were analysed by Western blotting on 15% SDS-PAGE. Membranes were probed with a polyclonal antibody recognizing PSP/reg. Tubulin served as a loading control. **(C, D)** Quantification of PSP/reg protein levels by densitometry in cells treated with doxycycline as indicated. Western blots were analysed as described in the Materials and methods section and normalised to control. Data shown represent mean ± SEM from three independent experiments. *indicates p < 0.05 compared with untreated controls **(E, F)**.

We next investigated whether suppression of HNF1A and HNF4A function had a differential effect on *PSP/reg* mRNA induction. As reported previously [24], the inducible expression of the DN-HNF1A mutant significantly elevated *PSP/reg* mRNA (Figure 3A). Interestingly, suppression of HNF4A function by induction of the DN-HNF4A mutant led to a significant suppression of *PSP/reg* mRNA levels (Figure 3B), demonstrating that HNF4A negatively regulates *PSP/reg* mRNA expression. Western blot analysis indicated similar changes in PSP/reg levels occurred at the protein level (Figures 3C and 3D). Densitometric analysis of PSP/reg protein levels confirmed that there was a significant threefold increase in PSP/reg levels following overexpression of DN-HNF1A (for 48 h), and a significant decrease in PSP/reg levels following overexpression of DN-HNF4A (Figures 3E and 3F).

### Inhibition of HNF1A, but not HNF4A function decreases *crp* expression in INS-1 insulinoma cells

**Figure 4:**
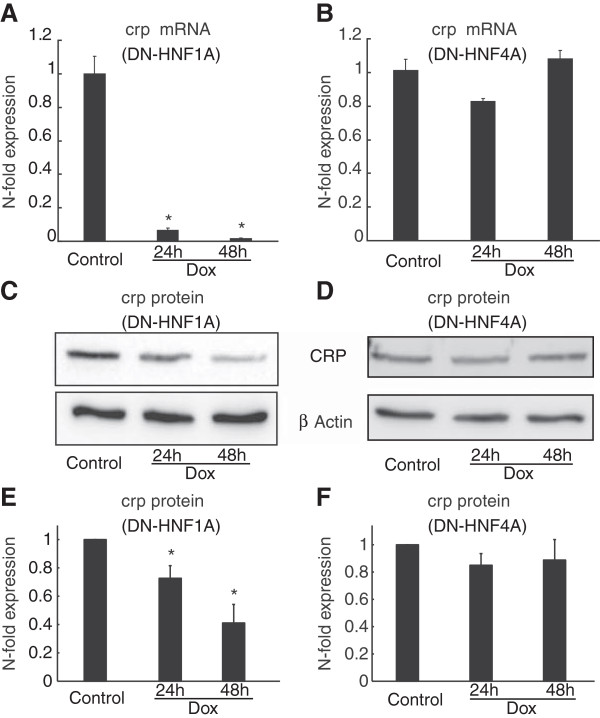
**Suppression of HNF1A, but not HNF4A function leads to a loss of *crp *expression.** DN-HNF1A and DN-HNF4A expression was induced in INS-1 cells by treatment with 500 ng/ml of doxycycline for 24 and 48 h as indicated. Cells were harvested and total RNA isolated. **(A-B)***crp* mRNA expression was determined using quantitative real-time PCR following normalization to β-actin. Data are represented as means ± SEM from *n* = 3 cultures. The experiment was repeated 3 times with similar results.* *p < 0.05* indicates the difference from non-induced controls. **(C-D)** Whole cell lysates were analysed by Western blotting on 15% SDS-PAGE. Membranes were probed with an anti-CRP polyclonal antibody. Antibodies raised against β-actin served as a loading control. **(E-F)** Quantification of CRP protein levels by densitometry in cells treated with doxycycline as indicated. Western blots were analysed as described in the Materials and methods section. Data shown represent mean ± SEM from three independent experiments. *indicates p < 0.05 compared with untreated controls.

Similar to our clinical observation of altered serum levels of PSP/reg1A in HNF1A vs. HNF4A-MODY subjects, serum levels of hsCRP have been suggested to be reduced in HNF1A- when compared to HNF4A-MODY subjects [29,30]. To explore whether we were able to detect a similar differential effect of HNF1A and HNF4A suppression on *crp* gene expression, we next determined *crp* mRNA levels in INS-1 cells inducibly expressing the HNF1A and HNF4A dominant-negative mutants. As expected from earlier studies demonstrating that *crp* is a HNF1A target gene [26], suppression of HNF1A function led to a potent down regulation of *crp* mRNA (Figure 4A). Interestingly, qPCR analysis of INS-1 cells with a suppressed HNF4A function demonstrated no change in *crp* mRNA expression (Figure 4B). Western blot analysis indicated similar changes in CRP levels occurred at the protein level (Figure 4C and 4D). Densitometric analysis of CRP protein levels confirmed that there was a significant decrease in CRP levels following overexpression of DN-HNF1A (for 24 h or 48 h), while CRP levels were not significantly affected while following overexpression of DN-HNF4A (Figures 4E and 4F).

### hsCRP and PSP/reg1A serum levels may discriminate HNF1A-MODY and HNF4A-MODY subjects

**Figure 5:**
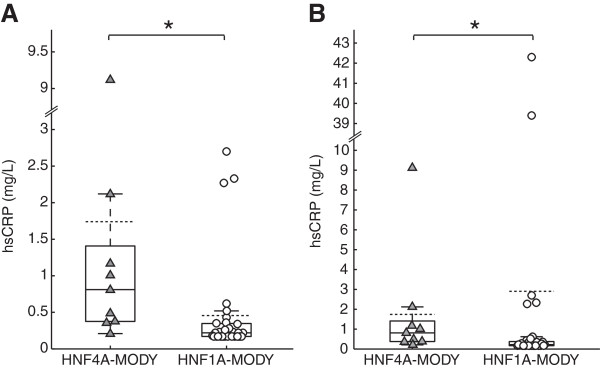
**Serum levels of hsCRP in HNF4A-MODY (▲) versus HNF1A-MODY (○).** Solid lines/box plot showing median and interquartile range and dotted line showing mean. **(A)** Serum levels excluding two subjects with extreme hsCRP levels. Median of hsCRP in HNF4A-MODY is 0.81 mg/l (IQR = 0.38-1.41 mg/l, n = 9), while median in the. HNF1A-MODY (IQR = 0.17-0.35 mg/l, n = 31) is 0.22 mg/l. The distributions differ significantly (*): Mann–Whitney U-test p = 0.002. **(B)** Including all hsCRP levels into analysis, increases IQR in HNF1A-MODY (median = 0.22 mg/l, IQR = 0.17-0.38 mg/l, n = 33). There is a significant difference in medians between HNF1A- and HNF4A-MODY (*): Mann–Whitney U-test p = 0.008.

Having identified two unique target genes of HNF1A and HNF4A that are differentially controlled by these two transcription factors, we next determined whether combined measurement of PSP/reg1A and hsCRP levels was able to discriminate HNF1A from HNF4A subjects. There was no correlation between hsCRP and HbA_1c_ levels in HNF1A-MODY (rho = 0.29, *P* = 0.1) and HNF4A-MODY subjects (rho = −0.05, *P* = 0.91). In agreement with previous studies, we found that serum hsCRP levels were significantly lower in HNF1A-MODY patients compared to HNF4A-MODY patients. Serum hsCRP levels of HNF1A-MODY patients after exclusion of those with hsCRP levels >10 mg/L were 0.22 (0.17-0.35) mg/L compared to HNF4A-MODY group [0.81 (0.38-1.41) mg/L, U-test *P* = 0.002] (Figure 5A). Serum hsCRP levels including all HNF1A-MODY patients were 0.22 (0.17-0.38) mg/L and also significantly lower [*P* = 0.008] compared to HNF4A-MODY (Figure 5B). However in both cases, there was a significant overlap of hsCRP values (Figure 5).

We therefore finally explored in a preliminary statistical analysis whether combined analysis of PSP/reg1A and hsCRP serum levels could increase the ability of the individual serum markers to discriminate HNF1A from HNF4A patients. ROC analysis on all data with the exclusion of the two patients with extreme hsCRP levels demonstrated that PSP/reg1A showed 90% sensitivity in identifying HNF1A-MODY patients, but with reduced specificity (67%) (Additional file 1: Figure S1B). In contrast, hsCRP showed a specificity of 89% in identifying HNF1A-MODY patients, at reduced sensitivity (77%) though. However when we performed a combined analysis of PSP/reg1A and hsCRP serum levels (such as the ratio of PSP/reg1A and hsCRP), we achieved the best sensitivity and specificity (84%, 26 out of 31 HNF1A and 89%, 8 out of 9 HNF4A, respectively) (Table 2) (Additional file 1: Figure S1B). Similar data were calculated when the ROC analysis was performed on all subjects’ data including those with high hsCRP values (Additional file 2: Table S1 and Additional file 1: Figure S1A).

**Table 2:** Performance of PSP/reg1A and hsCRP as individual or combined classifiers to distinguish HNF1A- from HNF4A-MODY

**Classification rule**	**Sensitivity**	**Specificity**
ROC: HNF1A if PSP > 9.34 ng/ml	90%	67%
ROC: HNF1A if CRP < 0.36 mg/L	77%	89%
ROC: HNF1A if PSP/CRP > 0.03	84%	89%
LDA: HNF1A if 1.25 - 0.10*PSP < 0	45%	78%
LDA: HNF1A if −0.69 + 0.63*CRP < 0	90%	33%
LDA: HNF1A if 1.17 - 28.25*PSP/CRP < 0	65%	89%

This data excludes the two subjects with extreme hsCRP levels. Classification rule shows the statistical method and the final rule to determine HNF1A-MODY. Sensitivity gives the percentage of correctly identified HNF1A-MODY; specificity shows the percentage of correctly identified HNF4A-MODY.

We validated these findings by calculating cut-off functions using LDA (Additional file 3: Figure S2). Investigating the discriminatory performance of PSP/reg1A and hsCRP as individual classifiers to distinguish HNF1A- from HNF4A-MODY subjects, excluding those with extreme hsCRP, we found PSP/reg1A to show 45% sensitivity and 67% specificity. The performance of hsCRP reached a sensitivity of 90%, however correctly detecting HNF4A-MODY was low with a specificity of 33%. LDA confirmed results from ROC analysis showing that a combination of both serum markers (for instance the ratio of PSP/reg1A to hsCRP) resulted in best LDA classification of 65% sensitivity (20 out of 31 HNF1A) and 89% specificity (8 out of 9 HNF4A) (Table 2). Performing LDA on all patient data resulted in similar classification qualities (Additional file 2).

HNF1A-MODY subjects showed significantly higher PSP/CRP ratio than HNF4A-MODY subjects [0.06 (0.03-0.08) vs. 0.01 (0.01-0.02), *P* = 0.001]. When HNF1A-MODY subjects with extreme hsCRP levels were included, the ratio is significantly higher (*P* = 0.003). The median and IQR of PSP/CRP ratio was unaffected. Further, we investigated the effects of mutation types. In this study, all mutations in HNF4A were missense mutations while only 4 HNF1A-MODY subjects had missense mutations with the remaining 29 subjects having truncating mutations. We found no difference between the PSP/CRP ratio in HNF1A-MODY subjects and mutation type [PSP/CRP ratio in subjects with missense mutations was 0.015 (0.002-0.069) vs. truncation mutations 0.062 (0.032-0.077), *P* = 0.13]. When the 2 patients with hsCRP >10 mg/L were excluded, one with a missense mutation and one with a truncation mutation, median PSP/CRP ratio of missense mutations was 0.025 (0.010-0.092) and of truncation mutations ratio is 0.063 (0.037-0.079). Again, we found no significant increase in the PSP/CRP ratio with more severe inactivating truncation mutations in HNF1A-MODY (*P* = 0.33).

## Discussion

The present study provides molecular proof and preliminary clinical proof-of-concept that the combination of serum PSP/reg1A and hsCRP levels may be of clinical use in distinguishing HNF1A-MODY from HNF4A-MODY subjects. Molecular validation of these clinical findings validated that HNF1A suppression negatively regulates *crp* mRNA and CRP protein levels in INS-1 cells, while *crp* gene and protein expression was normal in cells with a suppressed HNF4A function. Conversely, the induction of *PSP/reg* was inhibited by HNF4A suppression, but was not sensitive to HNF1A suppression. However it should be noted that overexpression of DN-HNF4A may also alter the activity of endogenous HNF1A**.**

Current guidelines for the genetic diagnosis of MODY recommend to test for *HNF1A* mutations if there is a history of young-onset diabetes before 25 years old in at least one family member, family history of diabetes (at least two generations), in the absence of pancreatic islet autoantibodies and without the evidence of insulin resistance [44]. Testing for *HNF4A* mutations is recommended when *HNF1A* genetic analysis does not show a mutation in individuals with clinical features of MODY or in diabetic family members with macrosomia or diazoxide-responsive neonatal hyperinsulinism [44]. Nevertheless, a recent study from the UK estimated that more than 80% of MODY cases are not diagnosed by molecular testing [45].

Barriers to molecular genetic testing include low availability and high financial cost of genetic testing. The availability of biomarkers which can be used in combination with clinical characteristics will enable clinicians to better identify cases of MODY and/or prioritise DNA testing. Though DNA sequencing is the ultimate proof of a genetic mutation, which cannot currently be substituted by any other test, measurement of PSA/reg1A and hsCRP levels can be performed to help the clinicians to correctly and rapidly identify patients with either HNF1A- or HNF4A-MODY and to aim for genetic counseling. Our proof-of-concept study suggests that parallel measurements of serum PSP/reg1A and hsCRP levels may be able to discriminate HNF1A- and HNF4A-MODY subjects with high confidence. In this study, we have demonstrated that HNF4A-MODY patients showed significantly lower levels of serum PSP/reg1A and significantly higher serum hsCRP levels compared to HNF1A-MODY patients. Our findings support the previous reported value of hsCRP as a diagnostic biomarker for HNF1A-MODY vs. HNF4A-MODY [28-30]. However the above cited earlier studies observed a significant overlap in hsCRP levels between these two groups. Therefore, the use of hsCRP as a marker to identify HNF1A-MODY may yield a high false positive rate. In addition hsCRP is a major acute-phase plasma protein which undergoes a rapid and marked rise of its serum concentration in response to infection or tissue injury [46]. In previous studies up to 10% of subjects had hsCRP levels higher 10 mg/L [29,30]. Serial hsCRP measurements would then be required after resolution of the infection. It has been demonstrated that there may be a positive correlation between hsCRP and HbA_1c_ when HbA_1c_ levels are > 9% in type 2 diabetic subjects [47]. In our study, there is a significant difference in HbA_1c_ levels between HNF1A-MODY and HNF4A-MODY subjects even though both groups had relatively low HbA_1c_ levels. HNF1A-MODY subjects had higher HbA_1c_ levels, yet their CRP levels were lower than HNF4A-MODY subjects. We found no association however between hsCRP and HbA_1c_ levels in HNF1A- and HNF4A-MODY subjects. It has been previously shown that there was no association between hsCRP and HbA_1c_ levels in HNF1A-MODY patients [28]. However, it is still possible that utilizing hsCRP as a biomarker alone for diagnosing HNF1A-MODY in a subject with an HbA_1c_ >9% may yield a false negative result.

Because of the routine availability of hsCRP testing, the clinical significance of hsCRP as a marker in identifying HNF1A-MODY subjects may become significant in the future. However, the combined detection of hsCRP and PSP/reg1A levels may provide an improved mean for better discrimination of HNF4A and HNF1A subjects.

In our study, we could not confirm PSP/reg and hsCRP to be normally distributed, which might be due to our small sample sizes in this proof-of-concept study. We applied ROC and LDA to investigate the usefulness of hsCRP and/or PSP/reg as biomarker to distinguish HNF1A- form HNF4A-MODY. Resulting classification rules are not optimal, and need to be confirmed in larger cohorts.

Identification of *HNF1A* and *HNF4A* mutation carriers has significant therapeutic implications as these subjects show sensitivity to low-dose sulphonylureas and subsequent alterations in treatment can improve glycaemic control in the majority of subjects [48]. HNF4A-MODY caused by mutations in the *HNF4A* gene is relatively less common than *HNF1A* mutations and accounts for approximately 5% of MODY cases worldwide [44]. However it is likely that many individuals with HNF4A-MODY remain undiagnosed. HNF4A-MODY subjects have similar progressive diabetic phenotype to HNF1A-MODY subjects, except that *HNF4A* mutations are associated with macrosomia, transient and persistent neonatal hypoglycaemia, later age of diabetes onset and the absence of low renal threshold [9,10]. It is crucial to identify *HNF4A* mutation carriers as heterozygous mutations in the *HNF4A* gene in either parent can impact on pregnancy. The offspring of *HNF4A* mutation carriers has a 50% chance of inheriting the mutation from either parent and has the risk of macrosomia due to increased insulin secretion in utero [11]. Therefore pre-pregnancy counseling for *HNF4A* mutation subjects is required and close monitoring during pregnancy and immediately at birth is needed to minimize complications of macrosomia and neonatal hypoglycaemia. The potential biomarkers to differentiate HNF4A-MODY from HNF1A-MODY would assist in better identification of HNF4A-MODY subjects and would enable these subjects to receive appropriate treatment and monitoring prior to and during the pregnancy to ensure successful maternal and neonatal outcome.

Of note, our study also confirms the clinical observation of a differential regulation of *crp* and *PSP/reg* genes by HNF1A and HNF4A on a molecular level. Previous studies have demonstrated that HNF4A- and HNF1A- regulated gene expression are remarkably similar [21-23], and that HNF4A acts upstream of HNF1A. Indeed there is evidence for a *cis-acting* element located upstream of the TATA box of the HNF1A promoter that has a high-affinity-binding site for HNF4A [14]. Previous studies have shown the presence of putative HNF1A binding elements within the promoter region of the *crp* gene, and a loss of *crp* expression was shown to be directly linked with altered regulation of HNF1A function [27,28,46]. Interestingly in our experimental studies we showed that INS-1 cells with an inducible suppression of HNF4A function showed no decrease in *crp* gene expression. In contrast, we observed a strong decrease in *PSP/reg* gene expression in response to suppression of HNF4A function, compared to a prominent induction of *PSP/reg* at the gene and protein levels during suppression of HNF1A function. Our study is therefore also one of the first reports that demonstrate differentially regulated HNF1A and HNF4A target genes.

## Conclusion

In conclusion, our study demonstrates that two distinct target genes, *PSP/reg* and *crp*, are differentially regulated by HNF1A and HNF4A. We have provided preliminary clinical proof-of-concept, and have mechanistically validated that the parallel measurements of serum PSP/reg1A and hsCRP levels in subjects with clinically suspected MODY may discriminate HNF1A-MODY from HNF4A-MODY. Our study therefore warrants clinical validation of these preliminary findings in a larger cohort of MODY subjects, as well as type 1 and type 2 diabetes patients.

## Abbreviations

HNF1A: Hepatocyte nuclear factor-1alpha; HNF4A: Hepatocyte nuclear factor-4alpha; MODY: Maturity-onset diabetes of the young; PSP/reg: Pancreatic stone protein / regenerating protein; hsCRP: High-sensitivity C-reactive protein; DN: Dominant-negative; ELISA: Enzyme-linked immunosorbent assay; Glut-2: Glucose transporter 2; INS-1: Insulinoma cell line-1; ROC: Receiver operating characteristic (ROC); LDA: Linear discriminant analysis.

## Competing interests

The authors declare that they have no competing interests.

## Authors’ contributions

MPK carried out recruitment and phenotyping of subjects, participated in the design of the study, statistical analysis, interpretation of data and drafting the manuscript. CB performed experiments in cellular models and participated in interpretation of data and drafting the manuscript. SB participated in recruitment and phenotyping of subjects. SMK performed experiments in cellular models and analysed data. JS performed statistical analysis and participated in manuscript drafting. RG measured PSP/reg and participated in the study design. JHMP supervised experiments in cellular models, participated in the design of the study, interpretation of data and manuscript drafting. MMB conceived of the study, participated in its design and coordination, supervised the study and drafted the manuscript. All authors read and approved the final manuscript.

## Supplementary Material

Additional file 1: Figure S1 Receiver operating characteristic (ROC) analysis testing all observed protein levels as potential thresholds to distinguish HNF1A- from HNF4A-MODY. A) Analysis of all subjects indicates ratio of PSP/CRP as the best marker based on the highest area under the curve (AUC = 0.82, green curve). HNF1A-MODY is predicted with sensitivity of 79% and specificity of 89% when PSP/CRP ratio >0.03 (threshold 3). CRP reaches AUC = 0.79 (blue curve) and predicts HNF1A with sensitivity 73% and specificity 89% when using threshold 2. PSP shows an AUC of 0.76 and high sensitivity of 90% with specificity of 67% when threshold 1 is used to distinguish HNF1A- from HNF4A-MODY. B) Excluding the two subjects with extreme CRP levels, ROC analysis shows similar results but for higher sensitivity. The combination of both proteins shows highest AUC = 0.88 with sensitivity for HNF1A of 84% and specificity to predict HNF4A of 89%. CRP reaches AUC = 0.84 and sensitivity = 77% with specificity = 89% at threshold 2. PSP shows an AUC of 0.75 with high sensitivity of 90% and specificity 67% when PSP > 9.34 ng/ml predicts HNF1A-MODY. Click here for file

Additional file 2 Performance of PSP/reg1A and hsCRP as individual or combined classifiers to distinguish HNF1A- from HNF4A-MODY. Click here for file

Additional file 3: Figure S2 Linear discriminant analysis (LDA) identifies thresholds to discriminate HNF1A- from HNF4A-MODY. Double lines on vertical axes represent disruption and change of scale. A) Analysis of all subjects (including two with extreme CRP levels, red) results in the best prediction quality for the combination of both markers. Predicting HNF1A when PSP/CRP ratio > 0.04 has a sensitivity of 64% and a specificity of 89%. Discriminating based on CRP alone reaches 89% to predict HNF4A but only 12% for HNF1A, using a CRP level of 3 mg/l as threshold. A threshold of PSP > 13 ng/ml achieves prediction of HNF1A with sensitivity 45% and specificity 78%. B) LDA of the data set excluding the two subjects with extreme CRP levels, finds similar results for analysis but for CRP as marker. As in A), combination performs best with specificity 89% and sensitivity 65% when PSP/CRP ratio >0.04 is predicted as HNF1A. CRP < 1.25 mg/l correctly predicts 90% of HNF1A subjects, but at the same time only 33% of HNF4A. Using PSP < 12.5 ng/ml as threshold discriminates 45% of HNF1A correctly from 78% of HNF4A subjects. Click here for file
