# Supplementary material for: Effects of hepatocyte nuclear factor-1A and -4A on pancreatic stone protein/regenerating protein and C-reactive protein gene expression: implications for maturity-onset diabetes of the young
Source: J Transl Med. 2013 Jun 26;11:156. doi: 10.1186/1479-5876-11-156 (PMC3707779; doi:10.1186/1479-5876-11-156)
Supplement: Additional file 1: Figure S1 — Receiver operating characteristic (ROC) analysis testing all observed protein levels as potential thresholds to distinguish HNF1A- from HNF4A-MODY. A) Analysis of all subjects indicates ratio of PSP/CRP as the best marker based on the highest area under the curve (AUC = 0.82, green curve). HNF1A-MODY is predicted with sensitivity of 79% and specificity of 89% when PSP/CRP ratio >0.03 (threshold 3). CRP reaches AUC = 0.79 (blue curve) and predicts HNF1A with sensitivity 73% and specificity 89% when using threshold 2. PSP shows an AUC of 0.76 and high sensitivity of 90% with specificity of 67% when threshold 1 is used to distinguish HNF1A- from HNF4A-MODY. B) Excluding the two subjects with extreme CRP levels, ROC analysis shows similar results but for higher sensitivity. The combination of both proteins shows highest AUC = 0.88 with sensitivity for HNF1A of 84% and specificity to predict HNF4A of 89%. CRP reaches AUC = 0.84 and sensitivity = 77% with specificity = 89% at threshold 2. PSP shows an AUC of 0.75 with high sensitivity of 90% and specificity 67% when PSP > 9.34 ng/ml predicts HNF1A-MODY. [file 1479-5876-11-156-S1.pdf]

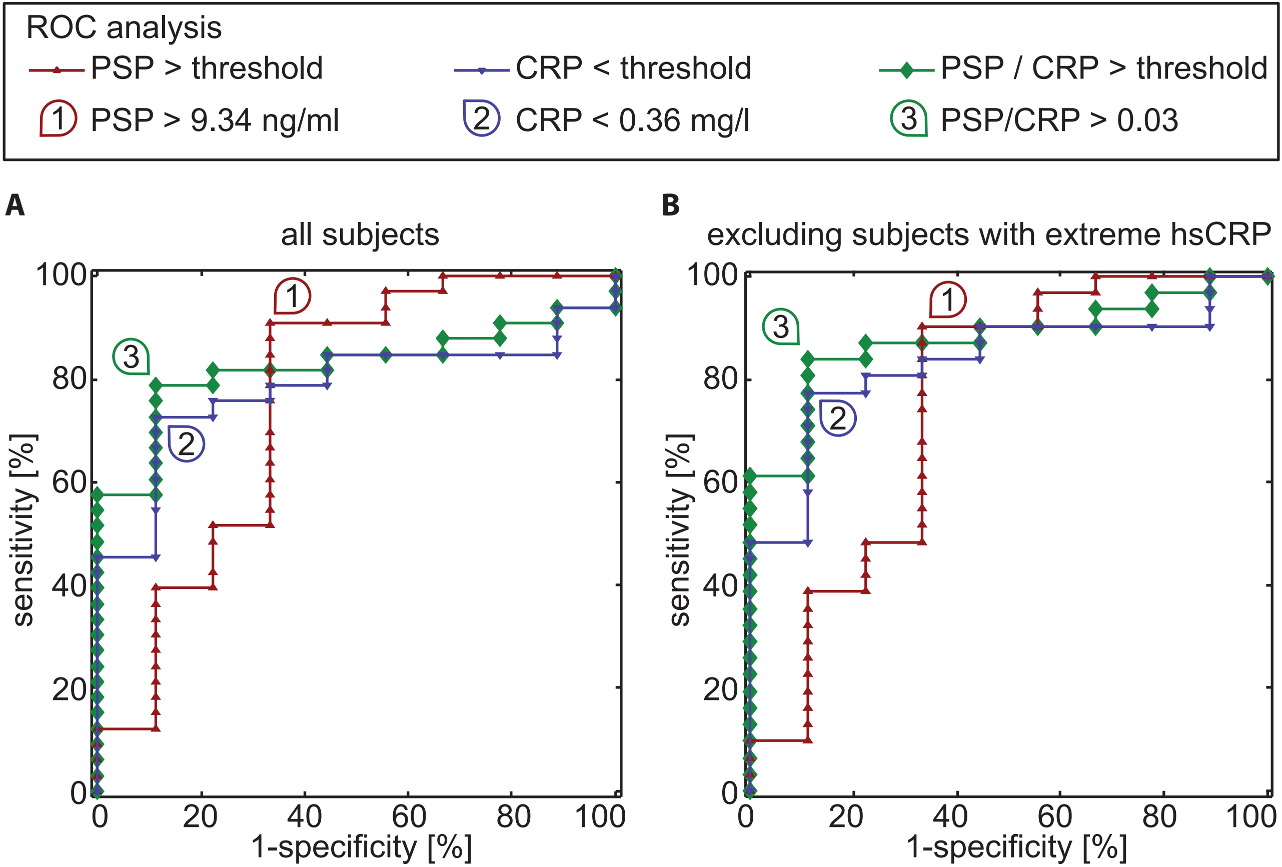
ROC analysis
PSP > threshold
CRP < threshold
PSP / CRP > threshold
1 PSP > 9.34 ng/ml
2 CRP < 0.36 mg/l
3 PSP/CRP > 0.03
A
all subjects
B
excluding subjects with extreme hsCRP
sensitivity [%]
1-specificity [%]
0
20
40
60
80
100
