# Supplementary material for: Effects of hepatocyte nuclear factor-1A and -4A on pancreatic stone protein/regenerating protein and C-reactive protein gene expression: implications for maturity-onset diabetes of the young
Source: J Transl Med. 2013 Jun 26;11:156. doi: 10.1186/1479-5876-11-156 (PMC3707779; doi:10.1186/1479-5876-11-156)
Supplement: Additional file 2 — Performance of PSP/reg1A and hsCRP as individual or combined classifiers to distinguish HNF1A- from HNF4A-MODY. [file 1479-5876-11-156-S2.pdf]

Supplementary Table. Performance of PSP/reg1A and hsCRP as individual or combined classifiers to distinguish HNF1A- from HNF4A-MODY

	**Sensitivity**	**Specificity**
**ROC: HNF1A if PSP > 9.34 ng/ml**	90 %	67 %
**ROC: HNF1A if CRP < 0.36 mg/L**	73 %	89 %
**ROC: HNF1A if PSP/CRP > 0.03**	79 %	89 %
**LDA: HNF1A if 1.30 - 0.10*PSP < 0**	42 %	78 %
**LDA: HNF1A if 0.03 – 0.01*CRP < 0**	12 %	89 %
**LDA: HNF1A if 0.96 – 24.28*PSP/CRP < 0**	64 %	89 %

This data includes all subjects. Classification rule shows the statistical method and the final rule to determine HNF1A-MODY. Sensitivity gives the percentage of correctly identified HNF1A-MODY; specificity shows the percentage of correctly identified HNF4A-MODY.
