# Supplementary figures and images for: Effects of hepatocyte nuclear factor-1A and -4A on pancreatic stone protein/regenerating protein and C-reactive protein gene expression: implications for maturity-onset diabetes of the young
Source: J Transl Med. 2013 Jun 26;11:156. doi: 10.1186/1479-5876-11-156 (PMC3707779; doi:10.1186/1479-5876-11-156)

**A** LDA analysis, all subjects

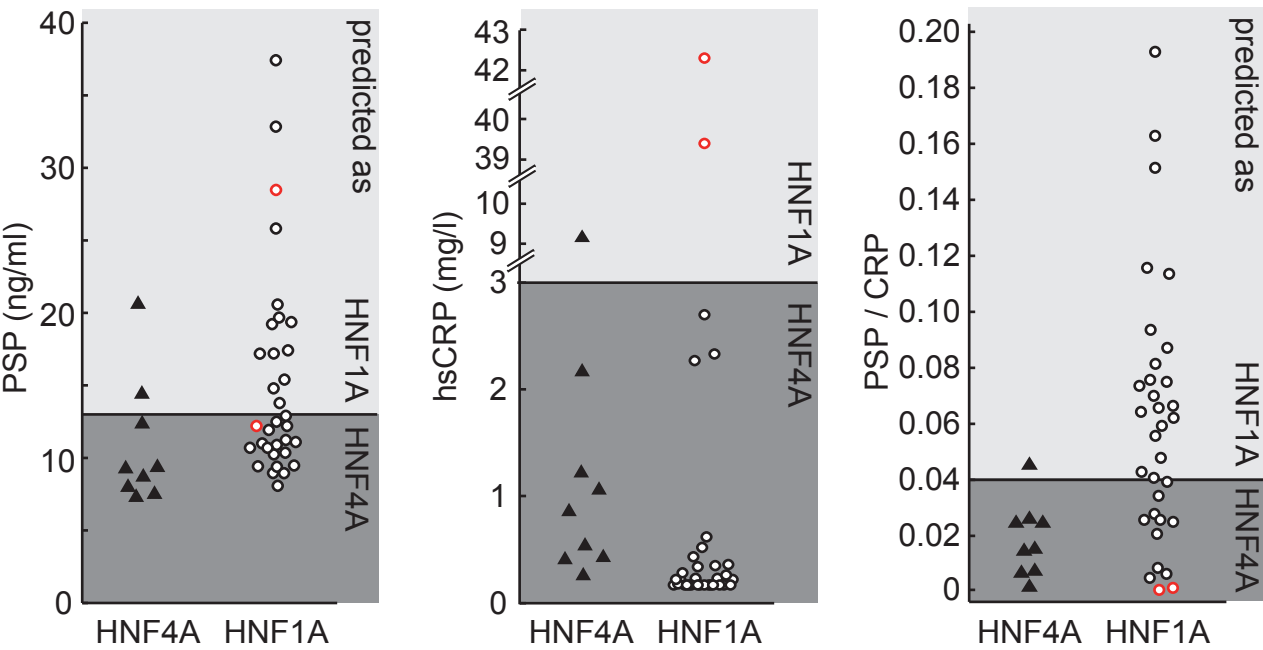

**B** LDA analysis, excluding subjects with extreme hsCRP levels

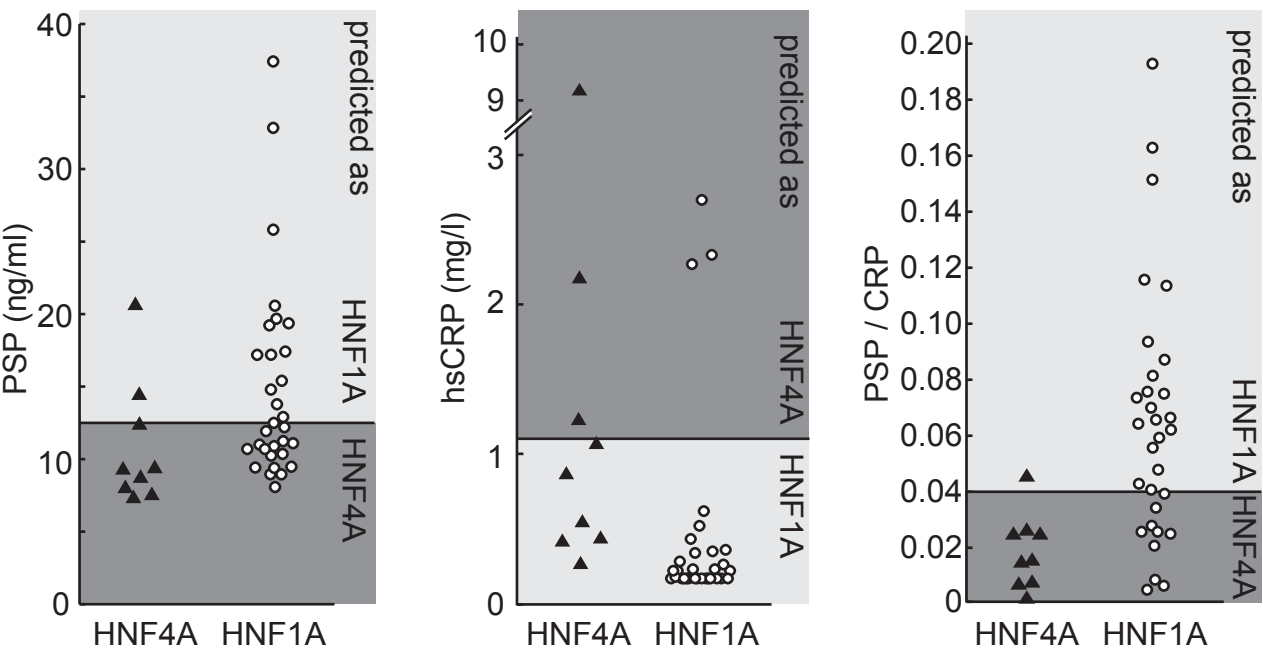

Supplement: Additional file 3: Figure S2 — Linear discriminant analysis (LDA) identifies thresholds to discriminate HNF1A- from HNF4A-MODY. Double lines on vertical axes represent disruption and change of scale. A) Analysis of all subjects (including two with extreme CRP levels, red) results in the best prediction quality for the combination of both markers. Predicting HNF1A when PSP/CRP ratio > 0.04 has a sensitivity of 64% and a specificity of 89%. Discriminating based on CRP alone reaches 89% to predict HNF4A but only 12% for HNF1A, using a CRP level of 3 mg/l as threshold. A threshold of PSP > 13 ng/ml achieves prediction of HNF1A with sensitivity 45% and specificity 78%. B) LDA of the data set excluding the two subjects with extreme CRP levels, finds similar results for analysis but for CRP as marker. As in A), combination performs best with specificity 89% and sensitivity 65% when PSP/CRP ratio >0.04 is predicted as HNF1A. CRP < 1.25 mg/l correctly predicts 90% of HNF1A subjects, but at the same time only 33% of HNF4A. Using PSP < 12.5 ng/ml as threshold discriminates 45% of HNF1A correctly from 78% of HNF4A subjects. [file 1479-5876-11-156-S3.pdf]
